# Digital Health Interventions to Promote Physical Activity in Community-Dwelling Older Adults: A Systematic Review and Semiquantitative Analysis

**DOI:** 10.3389/ijph.2024.1607720

**Published:** 2025-01-03

**Authors:** M. Di Pumpo, A. Miatton, M. T. Riccardi, E. A. Graps, V. Baldo, A. Buja, G. Damiani

**Affiliations:** ^1^ Department of Life Sciences and Public Health, Università Cattolica del Sacro Cuore, Rome, Italy; ^2^ Department of Prevention, AULSS6 Euganea, Padua, Italy; ^3^ Department of Cardiac, Thoracic, Vascular Sciences, and Public Health, Università degli Studi di Padova, Padua, Italy; ^4^ Cancer Screening Coordination Unit, Local Health Unit Roma 2, Rome, Italy; ^5^ Department of Biomedicine and Prevention, University of Rome “Tor Vergata”, Rome, Italy; ^6^ Agenzia Regionale per la Salute ed il Sociale ARESS, Bari, Italy; ^7^ Department of Woman and Child Health and Public Health, Fondazione Policlinico Universitario A. Gemelli IRCCS, Rome, Italy

**Keywords:** ageing, exercise, physical fitness, healthy lifestyle, digital health

## Abstract

**Objectives:**

Physical activity (PA) is crucial for older adults’ wellbeing. Digital health interventions (DHIs) are important, however a synthesis aimed at healthy community-dwelling OA is lacking. This study aims to synthesize DHIs effect on PA levels among community-dwelling 60-year-old adults or older.

**Methods:**

A systematic review was performed. DHIs using eHealth/mHealth tools, apps and text messaging were included. Primary outcomes were daily steps, moderate-to-vigorous PA and sedentary time. Quality was assessed via Cochrane risk-of-bias tools. Study-reported effect, study quality, sample size, study duration and dropout rate were semi-quantitatively synthesized to determine the overall category effect.

**Results:**

12 studies were included. 75% were low-quality, sample size was 16–18,080, study duration was 3–18 weeks, average dropout rate was 4.2%–46.7%. The synthesis of “motivational reminders” and “dynamic exercise programs” showed an overall positive effect, of “PA self-monitoring” showed mixed results and “exercise digital coaching” showed a non-positive effect.

**Discussion:**

Motivational reminders and dynamic exercise programs proved more effective in increasing PA in older adults than other interventions and should be more embedded in structured public health programs.

## Introduction

Physical activity (PA) is pivotal in maintaining the physical, mental, and social wellbeing of older adults (OA) [1, 2]. Regular PA effectively reduces the risk of chronic conditions [3], enhances cognitive function [4], fosters social interactions [5], and contributes to an improved quality of life (QoL) [6]. Walking, swimming, or yoga delays, for instance, the onset of cognitive impairment [7]. It fosters social connections, combating isolation in community settings [8]. Furthermore, PA is associated with improved psychological wellbeing, essential for QoL in OA [9]. For these reasons, the WHO (World Health Organization) aims to tackle this public health issue [10].

A significant proportion of OA remain inactive. Only a minority meets the WHO recommended levels [11, 12]. The prevalence of adults not meeting the recommended levels is 40%–60% among females aged 45–69 years and 50%–70% among females aged 70 years. In parallel, this prevalence is 30%–50% among males aged 45–69 years old and 40%–60% among 70-year-olds males and older across different WHO regions [12, 13].

In Europe, this prevalence among 55-year-olds and older varies widely, from 4.9% in Sweden to 29.0% in Portugal [14]. On average, OA spend around 9.4 h/day as sedentary [15]. This is often connected to retirement, fewer opportunities for social engagement, and a gradual decline in energy levels [16].

This poses a significant challenge, considering the proportion of people over 60 years old will nearly double by 2050 [17]. The positive effects of increased PA translate into a reduced disease and disability, with lower healthcare costs for chronic conditions management [18]. Moreover, regular PA extends life expectancy and also improves QoL by preserving independence, reducing the risk of disability, and promoting greater social engagement [19]. Digital health interventions (DHIs) emerge as promising opportunities for PA promotion among OA [20]. These interventions leverage information and communication technologies, such as websites, mobile applications, wearable devices, and telehealth platforms, to deliver personalized, scalable, and interactive PA interventions [21]. In 2017, Tedesco et al. reviewed the activity trackers for OA, detailing indicators like energy expenditure, activity assessment and sedentary behavior monitoring, all possible with commercially available devices [22]. The review emphasized the availability of several trackers specifically designed for OA, in addition to those for all ages. More evidence shows a growing interest in different trackers from major brands such as Fitbit and alike, used for interventions targeting the general population [23–25].

eHealth interventions have the potential to address various barriers faced by OA, including limited access to traditional exercise facilities [26]. The ever-increasing number of studies that analyze this public health aspect and the technologies involved corroborate its importance [20, 27–29] for health promotion strategy. However, a synthesis of DHIs aimed at healthy or with generic comorbidities community-dwelling OA is lacking.

The current study, therefore, aims to summarize the effect of DHIs interventions on PA levels of community-dwelling aged 60 years and older by a systematic review of the literature and semiquantitative analysis of the pertinent interventions from a health promotion and public health perspective. The objective is to provide evidence to guide public health policies, ensuring they align with the changing demographic landscape, evolving health needs, and technological advancements.

## Methods

The following PICO was adopted to guide studies inclusion:

- Population (P): community-dwelling OA (60 years of age or older);
- Intervention (I): digital communication tools or devices (eHealth/mHealth interventions; Digital interventions; Text-messaging; App-based interventions);
- Comparator (C): control group, self (in case of within-subject design), standard practice;
- Outcome (O): PA level, active lifestyle or sedentary behavior.

This study adopted the FDA (Food and Drug Administration) definition of digital health, which includes “mobile health (mHealth), health information technology (IT), wearable devices, telehealth and telemedicine, and personalized medicine” [25].

Only papers with the following characteristics were included:

- Randomized controlled trials (RCTs) or quasi-experimental studies comparing a Digital Health PA intervention targeting OA aged 60 years and older with a digital or non-digital PA intervention, or no intervention were considered for inclusion.
- English papers with full-text availability were considered for inclusion.
- Studies examining community-dwelling OA, either healthy or with multiple non-severe comorbidities (i.e., diabetes, hypertension etc.) not affecting in any way the ability to perform PA as compared to the general population.
- Studies reporting digital/mHealth/eHealth interventions of any duration to increase regular PA levels or to reduce sedentary behavior time were included, covering interventions using apps, automated text-messaging, digital reminders, and automated digital coaching.

Papers with the following characteristics were excluded:

- Studies with subjects in rehabilitation, hospital inpatients or residents in any healthcare setting. Considering how a consistently shared definition of “older age” was lacking in literature, 60 years of age was adopted as a cut-off point.
- Studies including telephone-based interventions, unless pre-registered and fully automated, or with the tools not accessible via computer or handheld devices (telephones, smartphones, or tablets).
- Studies reporting interventions either specific to a single disease and/or addressing whatever grade of physical inability to exercise (i.e., an exercise program without upper extremity activity to a patient with scapular girdle arthrosis).
- Studies providing participants with self-tracking devices to monitor activities without any health promotion components, such as coaching, support, or motivation.

Only the following were included as comparators:

- A within-subject baseline assessment of the same outcome measures before the intervention;
- An alternative intervention (featuring non-digital tools or other digital tools) or delayed intervention;
- A no-intervention control group.

With regards to primary outcomes, improved levels of regular PA or reductions in sedentary behaviors, such as increasing daily steps, reduction of sedentary time, and increasing the frequency of MVPA sessions were included. In particular, only studies with PA measures either tracked objectively through digital measurement devices or recorded subjectively through other forms of structured data analysis (e.g., via validated scales/tools; studies relying on open-ended questionnaires were excluded), were included. The effect measures were intended as relative and/or absolute differences in PA levels and other outcomes, expressed as numbers or percentages, depending on the study.

With regards to secondary outcomes, the following were included: changes in health parameters such as weight, glycated hemoglobin (HbA1c), cardiovascular health parameters, QoL and adherence rates.

With regards to literature search, the electronic databases PubMed (MEDLINE), Web of Science and Scopus, were queried by AM and MDP. An appropriate search string was created using Boolean operators to combine multiple synonyms related to the following topics: populations aged 60 and older, digital health, mobile apps, communication media, physical activity, and sedentary behavior (see Supplementary Material S1 for the search strings in their exact syntax). Only English papers of interventional studies published after 01-01-2000 were included. The search string was rerun prior to final analysis; however, no new suitable studies were found.

**FIGURE 1:**
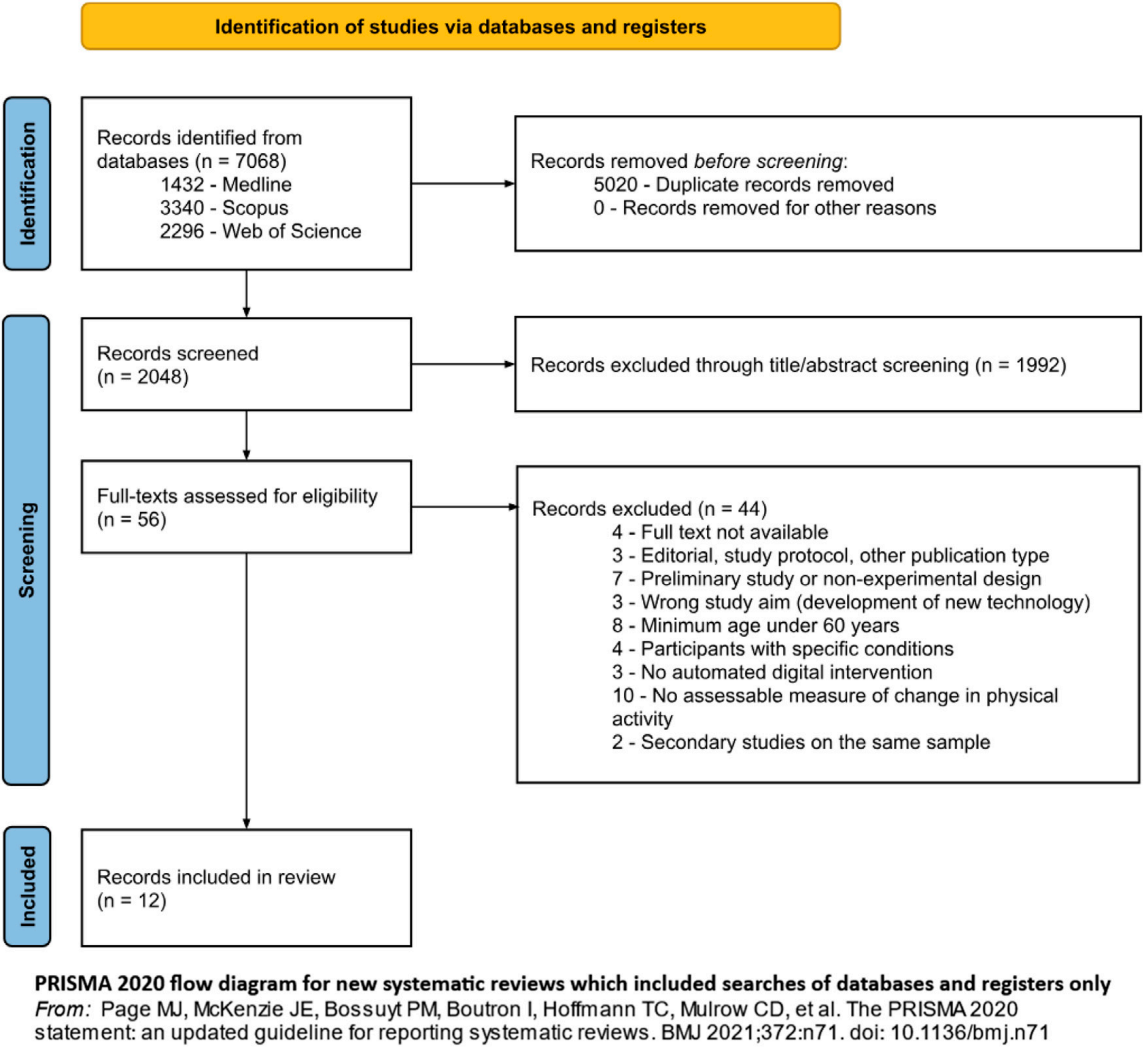
Flow diagram representing study selection and inclusion, from Page MJ et al. The PRISMA 2020 statement: an updated guideline for reporting systematic reviews. BMJ 2021; 372: n71. doi: 10.1136/bmj.n71 (Di Pumpo M. et al. Digital health interventions to promote physical activity in community-dwelling older adults: a systematic review and semiquantitative analysis. Padua, Italy. 2024).

With regards to study selection (see Figure 1), MDP and AM independently performed the process using Rayyan. Disagreements were resolved collectively.

After study selection, data extraction was performed by MDP and AM independently. Summary statistics were reported from original or performed following standard practice. No information relevant for synthesis was missing, no missing data handling techniques were required.

Regarding quality assessment, the included articles underwent a risk-of-bias (ROB) assessment by MDP and AM who independently applied the Cochrane tools. In detail, the Cochrane Rob2 tool was used for RCTs [30] and the Cochrane ROBINS-I tool [31] was used for non-randomized intervention studies. The tools’ final judgments were re-coded as high ROB (with “high risk” or “serious/critical” judgments obtained via the tools), medium ROB (“moderate” or “some concern” judgments obtained via the tools) and low ROB (with “low risk” judgments obtained via the tools) to render Rob2 and ROBINS-I tool overall judgments comparable. Among major items of ROB evaluation are the randomization process, deviation from intended interventions, missing outcome data, measurement of outcome, and selection of reported results. Among major items of ROBINS-I evaluation are confounding, participant selection, intervention classification, outcome measurement, missing data, reporting bias. Studies with a high ROB were classified as low quality, medium ROB as medium quality and high ROB as low quality (see Supplementary Material S2). This assessment subsequently constituted a key dimension of the evidence synthesis process.

Disagreements between MDP and AM were resolved collectively.

**FIGURE 2:**
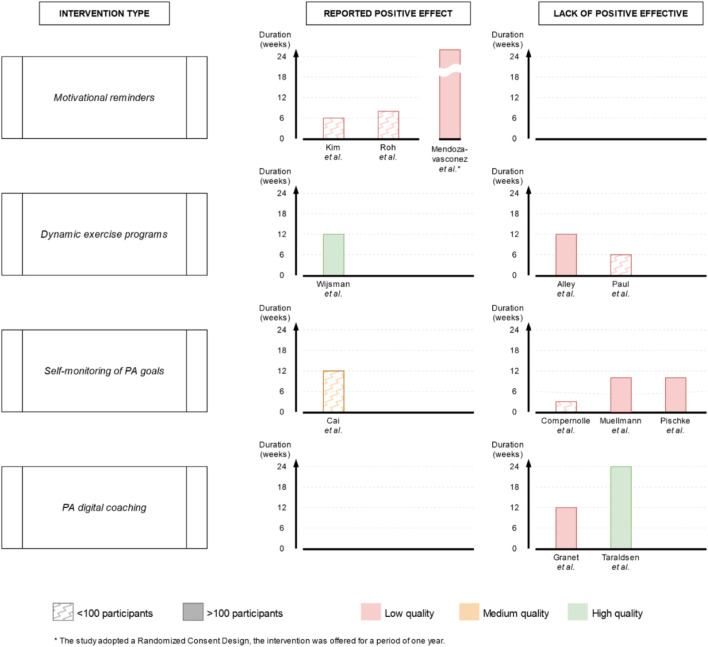
Harvest plot of intervention type by sample size, study quality, study duration and effect in increasing physical activity levels or reducing sedentary time (Di Pumpo M. et al. Digital health interventions to promote physical activity in community-dwelling older adults: a systematic review and semiquantitative analysis. Padua, Italy. 2024).

**FIGURE 3:**
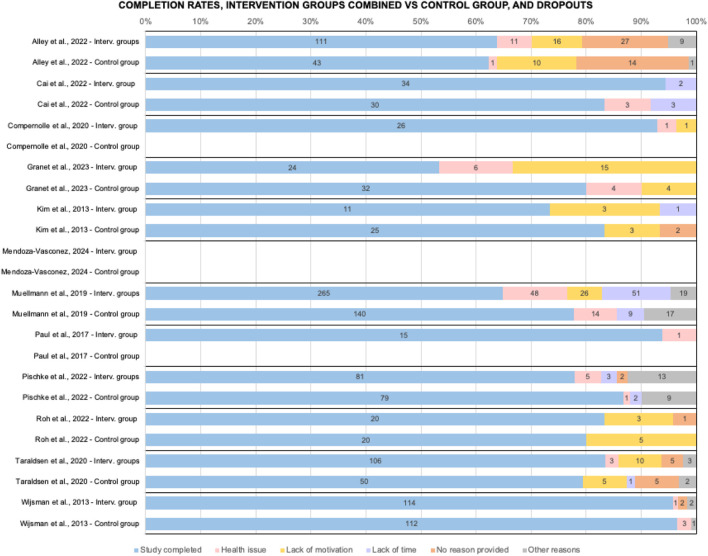
Comparison of studies on completion rates and number of dropouts by cause, interventions groups combined vs control group (when available) (Di Pumpo M. et al. Digital health interventions to promote physical activity in community-dwelling older adults: a systematic review and semiquantitative analysis. Padua, Italy. 2024).

Given the heterogeneity of data in terms of intervention types and recorded outcomes, a meta-analysis was deemed not feasible. Instead, a semiquantitative analysis was performed via a harvest plot in order to synthesize the effect on PA levels of the studied interventions, and inform the discussion and recommendations. In particular, the “reported effect” for each study was classified as “positive” the original study statistically significant enhancement in PA levels (step counts, MVPA, sedentary time, see Table 1), or as “non-positive” if otherwise. The intervention characteristics extracted were: reported effect, study quality as assessed via the quality assessment, sample size (classified as under or over 100 participants), study duration (in weeks), the number of participants who completed or abandoned the intervention and the reasons for this. These characteristics were compounded in the semiquantitative synthesis to determine the overall category “effect” and are graphically displayed in Figures 2, 3.

**TABLE 1:** Evidence table of the included studies (Padua, Italy. 2024).

Publication	Title	Study design	Context	Inclusion criteria	Exclusion criteria	Sample	Randomization	Intervention	Outcomes	Reported effect	Study assessment
*Main outcome measure: Daily step count*											
Cai et al., 2022 - European Geriatric Medicine [33]	Effects of peer support and mobile application-based walking program on physical activity and physical function in rural older adults: a cluster randomized controlled trial	Two-arm cluster RCT.	Hong Kong, between June 2017 and August 2018, in the community centers	a. Aged 65 years or older b. Able to walk independently with or without an assistive device c. Live in the community for at least 6 months d. Able to provide written informed consent	a. Have a medical condition that would make it unsafe to participate in physical activities b. Having cognitive impairment or mental health problems c. Be unable to communicate in Cantonese or read the study materials	72 participants: 46 females (63.9%), 26 males (36.1%) Mean age: 66.9 years (SD 4.2)	Intervention group (n = 36, mean age 65.8, SD 4.2), control group (n = 36, mean age 68.0, SD 4.1)	12 weeks Featuring: pedometer, group messaging app for smartphone (WeChat)	Step count (n/day) Other outcomes: physical activity self-efficacy, physical function (gait speed, grip strength, and chair-rising time), body composition (bioelectrical impedance analysis and dual-energy X-ray absorptiometry), and quality of life	Yes	Medium quality study (RoB 2: some concern). Quantitative PA outcome measures
Compernolle et al., 2020 - JMIR mHealth and uHealth [41]	Engagement, Acceptability, Usability, and Preliminary Efficacy of a Self-Monitoring Mobile Health Intervention to Reduce Sedentary Behavior in Belgian Older Adults: Mixed Methods Study	Mixed method pre/post design	Belgium (Flanders), from February to May 2019	a. Aged 60 years or older b. Able to speak Dutch c. Able to walk 100 m without severe difficulties d. Have a smartphone	-	28 participants: 15 females (53.6%), 13 males (46.4%) Mean age: 65.0 years (SD 4.6)	-	3 weeks Featuring: accelerometer with haptic feedback function (Activator by PAL Technologies), smartphone app for PA monitoring	Step count (n/day), total sedentary time (h/day) Other outcomes: sit-to-stand transitions, standing time, user engagement, acceptability, usability, satisfaction and perceived efficacy	No	Low quality study (ROBINS-I: Serious risk of bias). Quantitative PA outcome measures
Kim et al., 2013 - American Journal of Preventive Medicine [35]	Text Messaging to Motivate Walking in Older African Americans	Two-arm RCT.	USA (urban setting), between March and July 2011	a. Aged 60–85 years b. Community - dwelling c. American	a. Have any physical illness, psychological illness, or medical problems that restricted them from walking b. Not own a mobile phone with text messaging capability c. Not will- ing or able to follow study procedures	36 participants: 29 females (80.6%), 7 males (19.4%) Mean age: 69.7 years	Intervention group (n = 26, mean age 69.3, SD 7.3), control group (n = 10, mean age 70.6, SD 7.5)	6 weeks Featuring: pedometer (Omron, model #HJ-113), SMS-compatible mobile phone	Step count (n/day) Other outcomes: perceived activity levels (Leisure Time Exercise Questionnaire - LTEQ)	Yes	Low quality study (RoB 2: high risk) Quantitative and structured qualitative PA outcome measures
Paul et al., 2017 - Journal of Rehabilitation and Assistive Technologies Engineering [42]	Increasing physical activity in older adults using STARFISH, an interactive smartphone application (app); a pilot study	Mixed method pre/post design	Scotland (Glasgow), time period not specified	a. Aged over 65 years	-	16 participants: 8 females (50%), 8 males (50%) Mean age: 71.1 years (SD 5.2)	-	6 weeks Featuring: smartphone app with live wallpaper and pedometer functions (STARFISH)	Step count (n/day) Other outcomes: evaluation of the behavioral change techniques used, and app usability and acceptability	No	Low quality study (ROBINS-I: Serious risk of bias) Quantitative and non-structured qualitative PA outcome measures
*Main outcome measure: Moderate-to-vigorous physical activity*											
Alley et al., 2022 - Journal of Medical Internet Research [32]	The Effectiveness of a Computer-Tailored Web-Based Physical Activity Intervention Using Fitbit Activity Trackers in Older Adults (Active for Life): Randomized Controlled Trial	Three- arm RCT. Parent study: Active for Life (Alley et al., 2019)	Australia (Rockhampton, Bundaberg, and Adelaide), between 2018 and 2020	a. Aged 60 years or older b. Living independently in the community c. Able to provide informed consent	a. Have a medical condition that prevented them from engaging in physical activity b. Have a cognitive impairment that affected their ability to use technology c. Already meeting the physical activity guidelines d. Already using a Fitbit or other activity tracker	243 participants: 191 females (78.6%), 52 males (21.4%) Mean age: 69.5 years (SD 6.3)	Tailoring + Fitbit intervention group (n = 78, mean age 69.9, SD 4.1), tailoring-only intervention group (n = 96, mean age 69.1, SD 4.9), control group (n = 76, mean age 68.9, SD 3.9)	12 weeks Featuring: pedometer (Fitbit), web-based automated computer software	Moderate-to-Vigorous Physical Activity (min/week), sedentary time (min/day) Other outcomes: self-reported physical activity, self-reported sedentary time, quality of life, and social support	No	Low quality study (RoB 2 - high risk) Quantitative PA outcome measures
Muellmann et al., 2019 - Preventive Medicine Reports [36]	Effects of two web-based interventions promoting physical activity among older adults compared to a delayed intervention control group in Northwestern Germany: Results of the PROMOTE community-based intervention trial	Three-arm RCT. Parent study: PROMOTE (Muellmann et al., 2017)	Germany (Northwestern), from November 2015 to August 2018	a. Aged 65 or older b. No medical contraindications against physical activity c. Community - dwelling d. German speaking	a. Have a Mini-Mental-Score less than 25 b. Unable to participate in physical activity as assessed by the Physical Activity Readiness Questionnaire (PAR-Q) c. Could not provide written informed consent	529 participants: 299 females (56.5%), 230 males (43.5%) Mean age: 69.7 years	IG1 (web-based intervention only, n = 195, mean age 69.6, SD 3.4), IG2 (web-based intervention with accelerometer, n = 172, mean age 69.6, SD 3.2), CG (delayed control group, n = 162, mean age 69.8, SD 3.2)	10 weeks Featuring: accelerometer (ActiGraph GT3X+), web-based PA diary	Moderate-to-Vigorous Physical Activity (min/day) MVPA in-10-min-bouts (min/week), sedentary time (min/day), ST in-30-min-bouts (min/week)	No	Low quality study (RoB 2: high risk) Quantitative PA outcome measures
Pischke et al., 2022 - JMIR mHealth and uHealth [37]	Web-Based Versus Print-Based Physical Activity Intervention for Community-Dwelling Older Adults: Crossover Randomized Trial	Three-arm RCT. Parent study: PROMOTE (Muellmann et al., 2017)	Not specified (probably Germany, between 2019-2021), from October to February (for fall/winter sessions) and March to September (for spring/summer sessions)	a. Aged 60 years or older b. Live independently c. Provide informed consent	a. Have been physically active regularly for at least 2.5 h per week for >1 year b. Have participated in the previous trial c. Have planned vacation during the intervention period exceeding 2 weeks d. Be affected or been diagnosed with a medical condition that prohibits PA e. Present with severe visual or other impairments, implanted cardiac devices, or occasional syncopal episodes f. Present with cognitive impairment (MMSE-2-BV score <13)	204 participants: 135 females (66.2%), 69 males (33.8%) Mean age: 68.7 years (SD 5.4)	WEB group (n = 91, 67.9, SD 5.3), WEB+ group (n = 38, mean age 70.5, SD 6.0), PRINT group (n = 113, mean age 67.6, SD 4.9)	10 weeks Featuring: pedometer (Fitbit Zip), web- and app-based PA diary and digital coach	Moderate-to-vigorous physical activity (min/day), MVPA in-10-min-bouts (min/week), sedentary time (min/day), ST in-10-min-bouts (min/week) Other outcomes: acceptance, compliance, and reasons for discontinuation	No	Low quality study (RoB 2: high risk) Quantitative and non-structured qualitative PA outcome measures
Wijsman et al., 2013 - Journal of Medical Internet Research [40]	Effects of a Web-Based Intervention on Physical Activity and Metabolism in Older Adults: Randomized Controlled Trial	Two-arm RCT. Parent study: Philips DirectLife (Wijsman et al., 2013)	Netherlands (region of Leiden), from November 2011 to August 2012	a. Aged between 60 and 70 years b. Possession of personal computer with internet access c. Knowledge on how to use a personal computer	a. Active lifestyle as assessed by the General Practice Physical Activity Questionnaire (GPPAQ) b. History of diabetes or use of glucose lowering medication c. Physical inability or medical contraindication to increase physical activity level	235 participants: 96 females (40.9%), 139 males (59.1%) Mean age: 69.0 years	Intervention group (n = 119, mean age 64.7, SD 3.0), control group (n = 116, mean age 64.9, SD 2.8)	12 weeks Featuring: accelerometer (DirectLife - Tracmor triaxial accelerometer)	Moderate-to-vigorous physical activity (min/day), relative change in daily physical activity measured at wrist and ankle (%) Other outcomes: change in metabolic and anthropometric measures	Yes	High quality study (RoB 2: low risk) Quantitative PA outcome measures
*Other outcome measures of physical activity*											
Granet et al., 2023 - The Journals of Gerontology: Series A [34]	Web-Based Physical Activity Interventions Are Feasible and Beneficial Solutions to Prevent Physical and Mental Health Declines in Community-Dwelling Older Adults During Isolation Periods	Two-arm RCT.	Montreal, from April to August 2020 (COVDI-19 lockdown/homebound period)	a. Have an email address, an internet connection, and a digital device with a webcam at home b. Aged 60 years or older c. Live independently in the community d. Be inactive (less than 7.500 steps per day and less than 150 min of exercise per week) based on the Rapid Assessment of Physical Activity questionnaire (RAPA) e. Have no contraindication to practice PA.	a. Be frail (based on the study of osteoporotic fractures questionnaire) b. Use walking aids c. Be diagnosed with neurological, cardiovascular, lung (auto-reported) or have cognitive diseases/disorders (based on the Telephonic-Mini Mental State Examination [T-MMSE])	83 participants: 68 females (81.9%), 15 males (18.1%) Mean age: 70.1 years	Recorded Group (intervention group, n = 45, mean age 69.6, SD 5.1), Live Group (control group, n = 38, mean age 70.7, SD 5.2)	12 weeks Featuring: web-based or app-based video telephony software (Zoom)	Physical activity level (from the Rapid Assessment of Physical Activity questionnaire - RAPA) Other outcomes: feasibility and acceptability of the intervention, ability to use the technology, physical health outcomes, lifestyle habits, mental health, quality of life	No	Low quality study (RoB 2: high risk) Structured qualitative PA outcome measures
Mendoza- Vasconez et al., 2024 - American Journal of Health Promotion [43]	Engagement With Remote Delivery Channels in a Physical Activity Intervention for Senior Women in the US	Randomized Consent Design	USA (including urban, suburban, and rural settings), between November 2018 and November 2019	a. Being women b. 50–79 years of age c. Post menopausal d. If age 55, no menstrual period for at least 6 months; if age 50–54, no menstrual period for at least 12 months e. Intention to reside in area for at least 3 years	At time of enrollment: a. Lack of interest and/or signed consent b. Competing risk, including any medical condition with predicted survival of <3 years c. Safety reasons d. Adherence or retention reasons, including active participation in other randomized intervention trial	18,080 female participants (100%) Mean age: 83.1 years (SD 5.8)	-	Voluntary participation for a 1-year period Featuring various digital (email, study website) and non-digital (quarterly print newsletters, interactive voice response system - IVR) media to convey motivational messages	Total hours/week spent in all exercise-related activities, walking Metabolic Equivalents (from the Community Healthy Activities Model Program for Seniors - CHAMPS - questionnaire). Other outcomes: hours of sitting, engagement with four intervention channels (targeted printed newsletter inserts, emails, the WHISH website, and an IVR system)	Yes	Low quality study (RoB 2: high risk) Quantitative and non-structured qualitative PA outcome measures
Roh et al., 2022 – PLOSONE [38]	The effectiveness of a motivational enhancement smartphone application promoting lifestyle improvement for brain health: A randomized controlled trial	Two-arm RCT	Republic of Korea (Ajou University Hospital), over a non-specified 8-week period	a. Aged 60 years and older b. Using Android phone with an internet connection c. Literacy and a reliable informant to provide information to investigators	a. Any conditions preventing cooperation or interfering with the study, including history of psychiatric disorders, diagnosis of dementia, neurodegenerative diseases, serious medical conditions	40 participants: 26 females (65%), 14 males (35%) Mean age: 73.3 years (66.8-79.8)	Experimental group (n = 20, mean age 69.7), control group (n = 20, mean age 76.9, p-value<0.001)	8 weeks Featuring: motivational smartphone application	Moderate METs, vigorous METs (from the K-GPAQ), number of physical activity completion days per week (n/week) Other outcomes: brain health behavior in the categories of physical activity, cognitive activity, and a healthy diet.	Yes	Low quality study (RoB 2: high risk) Quantitative and structured qualitative PA outcome measures
Taraldsen et al., 2020 - Frontiers in Digital Health [39]	Digital Technology to Deliver a Lifestyle-Integrated Exercise Intervention in Young Seniors—The PreventIT Feasibility Randomized Controlled Trial	Three-arm RCT. Parent study: LiFE (Clemson et al., 2012)	Norway (Trondheim), Germany (Stuttgart) and Netherlands (Amsterdam), from March 2017 until August 2018	a. Aged 61–70 years b. Retired or working part-time c. Community dwelling (living independently) d. Able to read a newspaper or text on a smartphone e. Speak Norwegian/German/Dutch f. Able to walk 500 m without a walking aid g. Available for home visits during the following 6 weeks	a. Already participating in an organized exercise class (>1/week) b. Undertaking moderate-intensity physical activity (≥150 min/week in the previous 3 months) c. Have long-term travel plans (>2 months) within the next 6 months d. Have contraindication to exercise e. Have cognitive impairment (MoCA ≤24 points) or major depression (CES-D >24)	180 participants: 94 females (52.2%), 86 males (47.8%) Mean age: 66.3	eLiFE (n = 61, mean age 66.4, SD 2.3), aLiFE (n = 59, mean age 66.2, SD 2.3), controls (n = 60, mean age 66.4, SD 2.7)	24 weeks Featuring: digital coach app for smartphone and smartwatch (PreventIT)	Minutes of walking per day (%), adherence to recommendations (from the Exercise Adherence Reporting Scale - EARS) Other outcomes: physical behavior change, cognitive behavior change, healthy diet behavior, situational depression, motivation, readiness to change, adherence	No	High quality study (RoB 2: low risk) Structured qualitative PA outcome measures

## Results

A total of 7,068 records were identified (see Figure 1 for details).

Of the 12 included studies, 9 were RCTs (Alley, 2022 [32], Cai, 2022 [33], Granet, 2023 [34], Kim, 2013 [35], Muellmann, 2019 [36], Pischke, 2022 [37], Roh, 2022 [38], Taraldsen, 2020 [39], Wijsman, 2013 [40]), 2 were mixed-method studies with a pre/post design (Compernolle, 2020 [41], Paul, 2017 [42]), and one was a Randomized Consent Design study (Mendoza-Vasconez, 2024 [43]). A three-arm study design was used in 4 studies (Alley, 2022, Muellmann, 2019, Pischke, 2022, Taraldsen, 2020), comparing a web-based intervention with PA diary functions, an accelerometer combined with a web-based intervention, and a control group; a two-arm design was used in 5 other studies (Cai, 2022, Granet, 2023, Kim, 2013, Roh, 2022, Wijsman, 2013) comparing accelerometer/pedometer-based intervention with no intervention; finally, two studies (Compernolle, 2020, Paul, 2017) featured a pre/post study design and one study adopted a Randomized Consent Design (Mendoza-Vasconez, 2024).

At baseline, the 12 studies included a total sample size of 19,746, ranging from 16 (Paul, 2017) to 18,080 (Mendoza-Vasconez, 2024) participants, with a median sample size of 132 participants. Female participants ranged from 40.9% (Wijsman, 2013 - 96 females in 235 participants) to 100% (Mendoza-Vasconez, 2024). Mean age samples ranged from 65.0 (Compernolle, 2020) to 83.1 years (Mendoza-Vasconez, 2024). Study duration ranged from 3 weeks (Compernolle, 2020) to 6 months (Taraldsen, 2020), with most studies lasting 12 weeks (Alley, 2022, Cai, 2022, Granet, 2023, Wijsman, 2013), and one study being an extension of a community health study started in 1991 (Mendoza-Vasconez, 2024) [44]. 6 out of the 12 studies were conducted in Europe (Compernolle, 2020, Muellmann, 2019, Paul, 2017, Pischke, 2022, Taraldsen, 2020, Wijsman, 2013), 3 in Asia (Alley, 2022, Cai, 2022, Roh, 2022) and 3 in North America (Granet, 2023, Kim, 2013, Mendoza-Vasconez, 2024).

Among the outcomes considered in this systematic review, four studies assessed daily steps (Cai, 2022, Compernolle, 2020, Kim, 2013, Paul, 2017), and four other studies evaluated MVPA (Alley, 2022, Muellmann, 2019, Pischke, 2022, Wijsman, 2013). Additionally, of these eight studies, four also measured total sedentary time (Compernolle, 2020, Alley, 2022, Muellmann, 2019, Pischke, 2022). The remaining four studies included in the review used alternative methods to assess physical activity levels, including the Rapid Assessment of Physical Activity questionnaire (Granet, 2023), time per week spent in all exercise-related activities (Mendoza-Vasconez, 2024), the number of days per week on which the intervention has been completed (Roh, 2022), METs (Mendoza-Vasconez, 2024, Roh, 2022), time per day spent walking and the Exercise Adherence Reporting Scale (Taraldsen, 2020).

In 6 out of 12 studies (Alley, 2022, Cai, 2022, Compernolle, 2020, Muellmann, 2019, Paul, 2017, Pischke, 2022) PA was quantitatively measured using digital devices (i.e., accelerometers, pedometers, smartphone sensors), in 3 studies (Granet, 2023, Roh, 2022, Taraldsen, 2020) PA was estimated through structured qualitative tools (via validated questionnaires and/or as adherence to the intervention protocol), in 3 studies PA was assessed using both quantitative and structured qualitative tools (Kim, 2013, Mendoza-Vasconez, 2024, Wijsman, 2013). Out of the 12 included studies, 2 did not rely on objective PA outcomes assessment (Granet, 2023 and Taraldsen, 2020).

Quality of Life was estimated in 3 out of 12 studies through specific validated questionnaires (Cai, 2022, Granet, 2023, Taraldsen, 2020), however, none of these studies found a significant difference in quality of life between intervention and control groups. Feasibility, acceptability, usability, user engagement and/or satisfaction were examined in 7 out of 12 studies using ad-hoc questionnaires (Alley, 2022, Compernolle, 2020, Granet, 2023, Muellmann, 2019, Paul, 2017, Pischke, 2022, Taraldsen, 2020). However, none of these studies demonstrated significant positive results in increasing physical activity levels or reducing sedentary time.

Supplementary Material S3 shows in detail the main intervention instruments adopted in each study. Four main conceptually distinctive types of intervention were identified (as shown in Figure 2):

 •  Motivational reminders (n = 3; Kim, 2013, Mendoza-Vasconez, 2024, Roh, 2022): messages and reminders aimed at promoting and sustaining participants' active behaviors, sent exclusively through automated tools, either individually or to the entire intervention group;
•  Dynamic exercise programs (n = 3; Alley, 2022, Paul, 2022, Wijsman, 2013): tailored PA schedules made by an automated software using PA data gathered through a digital device, such as pedometers or accelerometers;
 •  Self-monitoring of PA goals (n = 4; Cai, 2022, Compernolle, 2020, Muellmann, 2019, Pischke, 2022): digital versions of a personal diary to record and monitor over time, with or without automatic data collection via digital device (e.g., pedometer, accelerometer) and peer support, the amount of physical activity performed and/or goals achieved;
 •  PA digital coaching (n = 2; Granet, 2023, Taraldsen, 2020): automated tools that provide instruction and guidance on performing physical exercises without the need for a human instructor available during training sessions.

A descriptive overview of the interventions for the promotion of physical activity in community-dwelling older adults, drawn from the studies included in the research, can be found as Supplementary Material S4.

Dropout rates ranged from 4.2% (Wijsman, 2013) to 46.7% (Granet, 2023). For larger studies average completion rate 72.6%, 79.3% in smaller studies (among controls was 81.7%). Excluding health problems (26.2% of dropouts from interventions), main causes were lack of motivation (25.5%), lack of time (19.7%) and others (15.9%).

Results related to secondary outcomes are shown in detail as Supplementary Material S5.

## Discussion

The semiquantitative synthesis identified different effects on PA levels with mixed characteristics. For the “motivational reminders” category, a positive effect was reported by three low-quality studies. For the “dynamic exercise programs” category results were mixed, with a positive effect reported by one high-quality study with high sample size versus two contrasting low-quality studies. For “digital PA coaching,” no positive effect was reported by one high-quality and one low-quality study. For the “self-monitoring of PA goals” category a no positive effect was reported by three low quality studies versus one contrasting medium quality study.

A first consideration is that the overall quality of the studies was found not sufficient, with 75% of the studies resulting as low-quality. This advocates for the importance of elevated methodological rigor.

In the category “motivational reminders,” all 3 studies (Kim, 2013, Mendoza-Vasconez, 2024, Roh, 2022) showed significant positive effect on physical activity levels of participants. This result is in line with literature [45, 46], which suggests that additional support provided in daily life, alongside exercise prescription, can be effective in promoting PA. No studies implemented goal-oriented functions, such as targets for physical activity or digital rewards. Instead, all three issued periodic reminders via non-personalized messages. Nor automation, facilitation nor coaching were provided. Participants manually filled in a personal diary for track. By contrast, supporting tools as wearable trackers (Kim, 2013, Mendoza-Vasconez, 2024), written brochures (Mendoza-Vasconez, 2024, Roh, 2022) and human assistance upon request were provided (Kim, 2013, Mendoza-Vasconez, 2024). Although promising, there are some concerns about reproducibility of the results regarding “motivational reminders.” The quality assessment revealed serious issues in all three studies, and the duration of the interventions was either short (Kim, 2013 - 6 weeks - and Roh, 2022 - 8 weeks) or inconsistent (Mendoza-Vasconez, 2024 - duration of participation up to each participant due to the randomized consent design of the study). Therefore, the semiquantitative evaluation tends to support a positive effect on physical activity levels of this type of intervention, nonetheless only further high-quality research will be able to draw definitive conclusions.

The results in the category “dynamic exercise programs” were mixed, with only 1 out of three interventions showing efficacy (Wijsman, 2013 on the one hand, Alley, 2022 and Paul, 2017 on the other). The features adopted in two interventions by Alley. (2022) and Wijsman 2013 included a wearable displaying dynamic goals and a digital PA diary synchronized with it. Paul (2017) also included digital rewards and peer messaging. However, the positive result was from a high-quality, two-arm RCT studying 235 participants for a reasonable time (3 months). In comparison, Paul (2017) adopted a pre/post study design with only 16 participants, while Alley (2022) adopted a three-arm study design comparing two different interventions (with or without the use of wearable technology) and a control group, showing the lowest completion rates among all the studies over 100 participants (63.8% intervention, 62.3% controls). This suggests that well-designed interventions leveraging wearable devices for automated goal setting and tracking can significantly enhance PA levels in OA. Fanning [47], who showed how tailored PA interventions can significantly improve health outcomes in OA. Bravata [48] demonstrated that pedometers are associated with significant increases in PA and improvements in health outcomes. App-based technologies, which are dynamic and can be adapted at any time to the user’s needs, are proving increasingly useful for the promotion of PA in elderly and intergenerational contexts [46].

The “self-monitoring of PA goals” category results were also mixed. A medium-quality study (Cai, 2022) showed efficacy in increasing daily steps in the elderly, while three other low-quality studies (Compernolle, 2020, Muellmann, 2019, Pischke, 2022) did not indicate any significant increase in daily moderate-to-vigorous physical activity or reduction in daily sedentary time. Besides the results of the quality assessment, one feature was unique to Cai’s study intervention in this category, namely, that participants were asked to manually fill in the digital diary to keep track of their progress. This approach is similar to what was observed for “motivational reminders.” Other tools provided by the intervention were written brochures to guide PA, peer support through a messaging app and in-person group sessions with trained professionals and peers. Given the study’s limited sample size (72 participants randomized into two groups) and the overall complexity of the intervention, its results cannot provide a reliable indication of effect for interventions that rely on self-monitoring of PA targets. As aforementioned, the self-monitoring aspect reportedly motivates health behaviors [49]. However, without proper engaging features, such as periodic motivational reminders or software designed to require the active participation of the elderly, personal digital exercise diaries may fail to maintain user interest or to significantly impact behaviors, as indicated by Webb [50].

Furthermore, both studies in the “PA digital coaching” category showed no evidence of efficacy (Garnet, 2023, Taraldsen, 2020). Furthermore, one of these studies (Taraldsen, 2020) conducted the longest intervention of all those included in this review (6 months), used an RCT design, included 180 participants and was deemed high quality. Therefore, it appears that digital coaching of exercises, especially when delivered through pre-recorded or on-demand videos, do not significantly improve physical activity levels in the elderly. This finding aligns with the literature suggesting that, while digital coaching can provide valuable information, it lacks the personalized and interactive components that drive behavior change. Morrison [51] highlighted the limited effectiveness of interventions without personalized feedback or interaction. These results show how both tailored exercise plans, dynamically adjusted via wearables, and peer-supported personalized apps result more promising than standalone digital coaching or personal digital diaries. This is in line with the shift towards operational, personalized instructions, especially when embedded in structured institutional programs [52].

Completion rates showed notable variability, with dropout rates largely influenced by low motivation and limited time available. Knowing the positive effects of physical activity appears to be insufficient to make OA prioritize it [53]. This underscores the importance of increasing their self-awareness about physical activity and encouraging them to take an active role for their health. Promoting autonomy and adaptability of interventions supports sustained behavior change and improve both completion rates and engagement [54].

In addition, it is important to underline how many studies (Cai, 2022, Muellmann, 2019, Paul, 2017, Roh, 2022) included a “peer support” component (as shown in Supplementary Material S3), which can be particularly effective when combined with motivational feedback. Indeed, Patel [55] reported increased activity levels in users receiving both peer support and personalized step goals. Digital peer support platforms facilitate information sharing and social support, which are key for major behavioral change theories [47] stating that self-awareness can motivate health behaviors. Maher [56] underscores the potential of social networks in increasing PA by providing social support and normative influence. As aforementioned, the self-monitoring aspect reportedly motivates health behaviors [49]. The difference in personalization dimension could help explain part of the difference in the reported effect between some of the included studies.

Finally, regarding secondary outcomes, engagement with motivational reminders varied significantly depending on factors like initial physical activity levels and functional status. Their general effectiveness on secondary health behaviors, like diet or cognitive activity, was limited. Dynamic exercise programs showed potential in reducing sedentary behavior and improving metabolic and physical health indicators. However, the success of these programs seemed dependent on structured guidance and accountability measures. Also, the preference for specific components suggests that these programs could benefit targeted modifications to fully address OA’s preferences. Self-monitoring tools were generally well-received, particularly for their usability and design. However, self-monitoring alone may not drive substantial behavior change or physical health improvements, as engagement levels varied widely, and usage declined over time. Integrating them with direct guidance and support is key. Digital coaching, in contrast with other synthesis findings, showed good results on motivation and adherence. Interactive and real-time coaching formats generated higher satisfaction and greater improvements in physical outcomes than non-interactive sessions, confirming how personalized interaction and social support plays an important role.

In a time of personalized health promotion policies seem to lag behind [57], and PA change is an excellent field to incorporate individual needs [58] via an increasingly more user-centered design [59]. This field will be greatly benefit from AI-based technologies [60].

These changes cannot be implemented without collaboration between professionals, public health institutional representatives and decision-makers in order to implement effective strategies. Public health initiatives should encourage the adoption of these technologies and also facilitate the partnerships and infrastructure necessary to support their widespread use [10]. It is crucial that all stakeholders are aligned to leverage these promising tools, ensuring they are integrated into comprehensive, equitable, sustainable, and engaging public health interventions.

The success of such interventions in a public health perspective is largely dependent on addressing barriers and facilitators to the use of these tools by OA [61], digital health literacy promotion [62, 63] addressing both individuals and professionals (however young or formally trained) [64], increasing the engagement of both individuals [65] and communities, especially if disadvantaged, by using evidence-based established frameworks [66].

This review needs to be considered in light of its limitations and strengths.

First, the application of the Risk-of-bias tool proved somewhat challenging, considering some criteria were not strictly suitable for the specific nature of the public health intervention studies.

Second, the total number of included studies was not large. However, the adopted stringent cut-off criterion for participant age ensured the inclusion of OA while effectively excluding middle-aged individuals, thereby enhancing the relevance and specificity of our findings to the targeted demographic. Furthermore, the inclusion of only community-dwelling OA, healthy or with generic multiple non-severe comorbidities not limiting their ability to participate in PA, made the results more comparable and scalable to population level.

A careful consideration must be made regarding Mendoza-Vasconez, 2024 due to significant differences both in study design (participants were included using a Randomized Consent Design from a large sample of 18,080 female participants) and intervention protocol (it consists of an analysis of an intervention delivered simultaneously through different means of communication over a long period of time). Its inclusion does not however change the considerations regarding the interventions it involves, considering all studies as shown by the harvest plot.

Another limitation of the study might be represented by the exploratory intervention classification performed. However, even though acknowledging important digital health intervention classifications in scientific literature [67], the study was already much focused in terms of PICO to specific targets, so that a general framework might have lost in applicability. This choice should therefore be regarded as a strength of the study, as it enabled the tailoring of concepts and interventions to the aim of the study and allowed the specific innovative technologies and techniques to emerge.

A major strength of the study is its focus on the most recent evidence-based research on community-dwelling adults aged 60 and over, with an emphasis on scalable, population-level technologies such as app-based tools, wearables and fitness trackers. These tools have the potential to be included in future health promotion intervention impacting many individuals, thanks to their extensive accessibility and widespread distribution. Smartphones and wearables may, in fact, be pivotal in decentralizing healthcare, allowing for health monitoring and telemedicine to become more widely accessible and integrated into daily life [68].

Finally, the current work is partially inspired by Muellman [69], however it presents substantial differences in population inclusion criteria (i.e., age cut-off, community setting, clinical conditions and health status), search strings, and data synthesis. The most interesting feature was the harvest plot. The authors believe it represents a major strength of this study, as it enables simultaneous evaluation of multiple key dimensions of all studies with a single illustration.

In conclusion, the present study synthesizing evidence regarding (DHIs) for community-dwelling OA addresses a significant gap in the scientific literature. The studies included in this review demonstrate considerable heterogeneity in both methodology and digital features. Nonetheless, comparative analysis allowed us to identify promising elements that could enhance physical activity in OA. Notably, dynamic, digitally-tailored exercise plans and periodic motivational reminders were part of the interventions showing positive effects on physical activity levels, likely due to their ability to empower participants and sustain motivation. Conversely, DHIs relying primarily on automation and facilitation were less effective in increasing physical activity and reducing sedentary time. As digital devices become increasingly accessible to OA, policymakers should consider evaluating this diverse array of DHIs to identify and implement the most evidence-based, effective health promotion interventions tailored to this demographic.

### Study Registration and Reporting

This systematic review is registered at PROSPERO (registration number: PROSPERO 2023 CRD42023470945. Available from: https://www.crd.york.ac.uk/prospero/display_record.php?ID=CRD42023470945). The research team followed the PRISMA guidelines (Preferred Reporting Items for Systematic Review and Meta-Analyses statement) for data search, extraction, synthesis and reporting.
