# Supplementary material for: Digital Health Interventions to Promote Physical Activity in Community-Dwelling Older Adults: A Systematic Review and Semiquantitative Analysis
Source: Int J Public Health. 2025 Jan 3;69:1607720. doi: 10.3389/ijph.2024.1607720 (PMC11738617; doi:10.3389/ijph.2024.1607720)
Supplement: Supplementary file 1 [file DataSheet2.PDF]

Supplementary file 2. Quality assessment

Rob-2 and ROBINS-I tool risk-of-bias assessment

STUDY NUMBER	STUDY REFERENCE	DOMAIN 1 *	DOMAIN 2	DOMAIN 3	DOMAIN 4	DOMAIN 5	OVERALL JUDGMENT (Rob2)
s1	Alley, S. J. et al. The Effectiveness of a Computer-Tailored Web-Based Physical Activity Intervention Using Fitbit Activity Trackers in Older Adults (Active for Life): Randomized Controlled Trial. J. Med. Internet Res. 24, e31352 (2022).	some concern	high risk	low risk	low risk	low risk	HIGH ROB
s2	Cai, X. et al. Effects of peer support and mobile application-based walking programme on physical activity and physical function in rural older adults: a cluster randomized controlled trial. Eur. Geriatr. Med. 13, 1187–1195 (2022).	some concern	low risk	low risk	low risk	low risk	MEDIUM ROB
s4	Granet, J. et al. Web-Based Physical Activity Interventions Are Feasible and Beneficial Solutions to Prevent Physical and Mental Health Declines in Community-Dwelling Older Adults During Isolation Periods. J. Gerontol. Ser. A 78, 535–544 (2023).	high risk	high risk	high risk	low risk	some concern	HIGH ROB
s5	Kim, B. H. & Glanz, K. Text Messaging to Motivate Walking in Older African Americans: A Randomized Controlled Trial. Am. J. Prev. Med. 44, 71–75 (2013).	some concern	high risk	high risk	some concern	high risk	HIGH ROB
s7	Muellmann, S. et al. Effects of two web-based interventions promoting physical activity among older adults compared to a delayed intervention control group in Northwestern Germany: Results of the PROMOTE community-based intervention trial. Prev. Med. Rep. 15, 100958 (2019).	some concern	some concern	high risk	low risk	some concern	HIGH ROB
s9	Pischke, C. R. et al. Web-Based Versus Print-Based Physical Activity Intervention for Community-Dwelling Older Adults: Crossover Randomized Trial. JMIR MHealth UHealth 10, e32212 (2022).	low risk	some concern	some concern	some concern	low risk	HIGH ROB
s11	Taraldsen, K. et al. Digital Technology to Deliver a Lifestyle-Integrated Exercise Intervention in Young Seniors—The PreventIT Feasibility Randomized Controlled Trial. Front. Digit. Health 2, (2020).	low risk	low risk	low risk	low risk	low risk	LOW ROB
s12	A. Wijsman, C. A. et al. Effects of a Web-Based Intervention on Physical Activity and Metabolism in Older Adults: Randomized Controlled Trial. J. Med. Internet Res. 15, e2843 (2013).<br>b. Vroege, D. P. et al. Dose-Response Effects of a Web-Based Physical Activity Program on Body Composition and Metabolic Health in Inactive Older Adults: Additional Analyses of a Randomized Controlled Trial. J. Med. Internet Res. 16, e3643 (2014).<br>c. "Broekhuizen, K. et al. An Internet-Based Physical Activity Intervention to Improve Quality of Life of Inactive Older Adults: A Randomized Controlled Trial. J. Med. Internet Res. 18, e4335 (2016)."	low risk	low risk	low risk	low risk	low risk	LOW ROB
s10	Roh, H.W. et al. The effectiveness of a motivational enhancement smartphone application promoting lifestyle improvement for brain health: A randomized controlled trial. PLoS One 17, e0267806 (2022). https://doi.org/10.1371/journal.pone.0267806.	some concern	some concern	some concern	some concern	some concern	HIGH ROB
s6	Mendoza-Vasconez, A.S. et al. Engagement With Remote Delivery Channels in a Physical Activity Intervention for Senior Women in the US. Am J Health Promot. Feb 12; https://doi.org/10.1177/08901171241229537 (2024). Epub ahead of print. PMID: 38344760	some concern	some concern	some concern	some concern	high risk	HIGH ROB

STUDY NUMBER	STUDY REFERENCE	DOMAIN 1 **	DOMAIN 2	DOMAIN 3	DOMAIN 4	DOMAIN 5	DOMAIN 6	DOMAIN 7	OVERALL ROB (ROBINS-I tool)
s3	Compernolle, S. et al. Engagement, Acceptability, Usability, and Preliminary Efficacy of a Self-Monitoring Mobile Health Intervention to Reduce Sedentary Behavior in Belgian Older Adults: Mixed Methods Study. JMIR MHealth UHealth 8, e18653 (2020).	serious	low risk	low risk	low risk	low risk	serious	moderate	SERIOUS
s8	Paul, L. et al. Increasing physical activity in older adults using STARFISH, an interactive smartphone application (app); a pilot study. J. Rehabil. Assist. Technol. Eng. 4, 2055668317696236 (2017).	serious	low risk	low risk	serious	serious	moderate	moderate	SERIOUS

*Rob2 tool domains:

DOMAIN 1: Bias arising from the randomization process;
DOMAIN 2: Bias due to deviations from intended interventions;
DOMAIN 3: Bias due to missing outcome data;
DOMAIN 4: Bias in measurement of the outcome;
DOMAIN 5: Bias in selection of the reported result.

**ROBINS-I tool domains:

DOMAIN 1: Bias due to confounding;
DOMAIN 2: Bias in selection of participants into the study;
DOMAIN 3: Bias in classification of interventions;
DOMAIN 4: Bias due to deviations from intended interventions;
DOMAIN 5: Bias due to missing data;
DOMAIN 6: Bias in measurement of outcomes;
DOMAIN 7: Bias in selection of the reported result.
