# Supplementary material for: Digital Health Interventions to Promote Physical Activity in Community-Dwelling Older Adults: A Systematic Review and Semiquantitative Analysis
Source: Int J Public Health. 2025 Jan 3;69:1607720. doi: 10.3389/ijph.2024.1607720 (PMC11738617; doi:10.3389/ijph.2024.1607720)
Supplement: Supplementary file 2 [file DataSheet4.PDF]

Supplementary file 4. Short evidence table

Summary of the characteristics of the included publications

Publication	Study aim	Recruitment	Description	Results	Conclusion
Alley et al., 2022 - Journal of Medical Internet Research	To evaluate the efficacy of a computer-tailored web-based physical activity intervention using Fitbit activity trackers in older adults.	Prospective participants were directed to the landing page of the intervention website through various channels, including social media, community groups, and local newspapers.	6 modules of computer-automated tailored advice based on participant data, delivered biweekly. Both tailoring+Fitbit and tailored advice only groups recived The Active for Life intervention, but Tailoring+Fitbit participants were also required to sync their Fitbit activity tracker with the website to measure their physical activity over the past 2 weeks, whereas the tailoring-only group answered a few additional questions that asked them to recall how many minutes of physical activity they had completed in the past 2 weeks. Participants in the delayed control group initially only received access to the research surveys. Participants recieved reminders and monetary incentives to complete all research surveys.<br>The advice is based on the theory of planned behavior and the social cognitive theory and includes evidence-based behavior change techniques, aimed at meeting the physical recommendations (30 minutes of moderate-intensity physical activity on at least 5 days each week, including 2 to 3 sessions of strength and flexibility activity), limit sedentary behaviour (<8 hours sitting time per day and regular breaks from sitting).	MVPA did not improve significantly in either intervention group, but decreased by 35% in the control group (time by group interaction just above the significance criterion). At week 12 a 43% difference in MVPA favored the tailoring+Fitbit group compared to the control group (sensitivity analysis). No overall time by group interaction for sedentary behavior measured by accelerometer or self-reported.<br>After the intervention, the tailoring+Fitbit and control groups significantly increased accelerometer-measured sedentary behavior compared with baseline (by 34 and 38 minutes per day, respectively, in sensitivity analysis), while the sitting time of the tailoring+Fitbit group decreased by 98 minutes but did not reach significance.<br>At week 6, week 12 and week 24, all groups increased self-reported physical activity compared with baseline (34% more in the tailoring+Fitbit group, but not significant) and participants in the tailoring+Fitbit group decreased self-reported sitting time.	Computer-tailored advice based on Fitbit measurement of physical activity in older adults is likely to lead to improved physical activity outcomes compared with no advice but not compared with advice based on self-reported physical activity. More research is needed to investigate ways to further improve effectiveness of computer-tailored advice based on Fitbit measurement in older adults.

Publication	Study aim	Recruitment	Description	Results	Conclusion
Cai et al., 2022 - European Geriatric Medicine	To evaluate the effects of a multicomponent physical activity intervention program on physical function, body composition, and quality of life of community-dwelling older adults in Hong Kong.	From community centers from 8 diferent villages in Hong Kong.	Participants in the intervention group received a 3-month peer support and mobile application-based walking programme. Participants in the intervention group received 30-minute, face-to-face physical activity motivation group sessions once a week for the first 2 weeks, a pedometer and a booklet containing advice on safe exercise, diaries for recording daily walking steps, and weekly exercise action planning forms. They were also taught how to use pedometers combined with the diary to record their steps, and instructed to set the goals for physical activity and to discuss the potential barriers to increase physical activity with their peers. Via the smartphone application named Wechat, intervention group participants could also share their activities and progress and receive advice on physical activity and behavior change techniques by research assistants. The control group only attended lectures during the first 2 weeks and received physical activity education and periodic support phone-calls. A total of 4 peers were recruited among retired community health workers, and instructed to do home visiting or make phone calls to participants once a week.	Compared with the control group, intervention group participants increased their pedometer-measured daily walking steps by 408.3 steps/day (P=0.03), with the effect size being 0.33 (95% CI 0.09 to 0.58). Physical activity self efficacy was improved with an efect size of 0.42 (95% CI 0.16–0.69) in the intervention group cmopared to the control group. Per-protocol analysis for these outcomes revealed comparable outcomes.	A multicomponent physical activity intervention program could be effective in improving physical function, body composition, and quality of life among community-dwelling older adults in Hong Kong.
Compernolle et al., 2020 - JMIR mHealth and uHealth	a. To gain insight into older adults' experiences with, and the use of, a self-monitoring–based mHealth intervention specifically developed to reduce sedentary behavior.<br>b. To get a first indication of the effect	Though an advertisement distributed via Facebook, and secondly, by electronically sending the advertisement to older adults who were included in a previous study by our research group and who had	General sedentary behavior information was provided to participants by means of a 10-minute presentation by an expert in the field during an home visit. During the intervention, participants were asked to wear the self-monitoring device (Activator, PAL Technologies). Real-time feedback and a 7-day historical overview on sedentary time, upright time, and number of steps were constantly available through a smartphone app (via Bluetooth connection to the device). Tactile feedback was provided by means of a strong, but comfortable, vibration of the Activator device itself	Small improvement in steps of around 400 per day, which was not significant probably because of the small sample size. Sitting and standing time were very similar at pre- and post- measurements. Participants commonly reported that the intervention changed their thinking (ie, they became more aware of their sedentary behavior) but not their actual sedentary behavior.	The intervention was generally well perceived by the elderly, but the intensity of the intervention was probably insufficient to reduce participants' sedentary behaviors. To achieve effective behavioral change, suggested modifications

Publication	Study aim	Recruitment	Description	Results	Conclusion
	size through assessment of sedentary behavior as preliminary efficacy of the intervention on older adults.	expressed interest in future studies. Convenience sampling was used until data were saturated (ie, until no new themes emerged in additional interviews).	each time a participant is sitting for 30 uninterrupted minutes, and repeated after another 30 minutes if a participant remained sedentary. Participants were able to turn the vibration function on and off. After the intervention, participants were instructed to wear the accelerometer for another week (ie, postmeasurements) and to complete a semistructured interview that included questions on user engagement with the intervention and perceptions regarding usability and acceptability.		to the intervention include adding behavior change techniques that target the controlled processes underlying sedentary behavior.
Granet et al., 2023 - The Journals of Gerontology : Series A	a. To confirm the feasibility and acceptability of 2 web-based PA interventions during the first COVID-19 lockdown.<br>b. To assess and compare the effects of two fully remote and tailored web-based PA interventions on physical and mental health in inactive community-dwelling older adults	From the CRIUGM's volunteer database, which contains a large number of community-dwelling older adults, who voluntarily registered to be invited to participate in research projects related to aging.	Three 55-minute sessions per week. The Live Group (control) sessions included a certified exercise instructor (kinesiologist; using Zoom) following a specific schedule (Monday, Wednesday, and Friday mornings), whereas the Recorded Group (intervention) attended individual video sessions, which were pre-recorded by a certified exercise instructor (kinesiologist). Every 4 weeks, the difficulty and intensity of the sessions were adjusted to maximize the health effects, adherence, and enjoyment. 4 levels of PA interventions (live vs recorded) were created (L1 = nonfit to L3 = fit) to tailor the exercise intervention for each participant. At baseline, a mobility decisional tree (6 tests to obtain a score from 0 to 14) was used to allocate partecipants to 3 exercise group levels (Level 1: score < 6; Level 2: 6 ≤ score ≥ 10; Level 3: score > 11).	PA levels from pre- to post- intervention increased for Live Group (from 4.5 ± 2.0 to 5.7 ± 2.6) and decreased for Recorded Group (from 4.5 ± 2.0 to 5.7 ± 2.6). PA habits were not significantly different in time- or group- comparisons, but the time by group interaction reached significance. Adherence was slightly higher and the drop-out rate was lower in the Live Group.	Live-interactive group interventions should be favored over the recorded modality. Recorded sessions may be appropriate for participants who cannot temporarily follow online and LG sessions, but exercise physiologists should perform careful follow-up when using this type of delivery to reduce dropouts. Both interventions were found to be feasible, acceptable, and safe for community-dwelling older adults during periods of isolation.

Publication	Study aim	Recruitment	Description	Results	Conclusion
Kim et al., 2013 - American Journal of Preventive Medicine	a. To examine the impact of using a motivational mobile text messaging intervention to increase step count among older community-dwelling African Americans.<br>b. To study the effects of text messaging on self-reported leisure-time exercise behavior.	In senior centers, staggered over time.	The intervention group received motivational text messages (modeled after those used in previous research on weight management with text messages) three times a day, 3 days a week. Both groups received pedometers and walking manuals to record step counts. Participants were not required to reply but had the option to reply with a comment or question. A private number was created by Google Voice for all study communications. For practice at the enrollment session, a test text message was sent to confirm that participants were competent in text messaging. If participants needed extra assistance, personal instructions for their mobile phones were given.	The group who received motivational text messages had greater improvements in step count (679 vs 398; $p<0.05$) and perceived activity levels ($p<0.05$) than the group who did not receive text messages.	Motivational text messaging three times a week for 6 weeks was effective in increasing step count and self-reported leisure time exercise behavior among older African Americans.
Mendoza-Vasconez, 2024 - American Journal of Health Promotion	To evaluate differences in PA among participants in the WHISH intervention arms and to identify the descriptive subgroup profiles of older women who actively engaged or did not engage with the different WHISH intervention channels.	Participants were recruited between 1993 and 1998 by 40 Clinical Centers in 24 states and the District of Columbia, using multiple recruitment strategies, with mass mailings being the primary method of identifying interested potential participants for initial screening.	At the beginning of the intervention, participants received a pedometer, paper calendars, resistance bands, and an exercise guide with examples of strength training exercises. Participants had the ability to set goals or track activity through paper calendars, the study website, or an interactive voice response system [IVR]. The intervention has continued to be delivered during subsequent years via different channels, including quarterly newsletters with targeted inserts for participants with lower, middle and higher levels of PA and PF; monthly outbound telephone calls with short (~1 minute) motivational messages; monthly motivational emails with links to vetted websites (for the approximately one-fourth of participants who provided email addresses); a website with additional resources including videos demonstrating exercises; and occasional personal contact with staff by phone, e-mail, or mail as required or requested.	Overall, 38% of WHISH intervention participants engaged with at least one of the WHISH intervention channels. All engagement variables were significantly positively associated with hrs/week of all exercise related activities (square root coefficients ranging from .29 for logging into the website, to .13 for opening emails in adjusted models). Additionally, all engagement variables, except for engagement with IVR, were positively significantly associated with walking METs (square root coefficients ranging from .29 for logging into the website, to .08 for opening emails). Conditional Inference Trees for all engagement models highlights that subgroups with the highest engagement are consistently younger, report higher early intervention PA or walking METS, higher quality of life and physical functioning, and lower cognitive change scores; additionally, groups with the highest engagement have higher proportions of individuals with a college degree or above, not using a walking aid, and with excellent general health.	Large-scale remotely-delivered PA programs may be viable and effective in attaining population-level impact. Never theless, these interventions should be deployed with caution to not exacerbate health disparities. It may be necessary to use a step-care approach or to more precisely target subgroups that are harder to engage.

Publication	Study aim	Recruitment	Description	Results	Conclusion
Muellmann, 2019 - Preventive Medicine Reports	To evaluate the effectiveness of two web-based interventions promoting physical activity among older adults in northwestern Germany compared to a delayed intervention control group.	Through advertisements and community contacts.	Participants in intervention group 1 received access to a web-based diary to track their PA behavior, participants in intervention group 2 additionally received a Fitbit Zip synchronized with the website. Both groups were provided with printed brochures outlining exercises for different difficulty levels and gender, as well as information about local PA offers in the respective community. The website provided weekly feedback on PA goals and specific goal-triggered rewards (digital cups). Participants had the opportunity to network via the website “invite friend” function and the online forum. Weekly group meetings by trained research assistants were also offered. The two web-based interventions were developed based on self-regulation theory and principles of behavior change. Participants assigned to the delayed control group received no intervention during the ten-week intervention.	Results were only significant for weekly MVPA in 10min bouts (F = 3.31, p = 0.04). At follow-up, total weekly MVPA time in 10-minute bouts and daily MVPA minutes decreased in IG1 participants and increased in IG2 participants, compared to baseline and CG participants. There were no significant changes in daily sedentary time, while weekly sedentary time in 30-minute bouts increased in IG1 at follow-up compared to baseline and CG participants. Differences in MVPA and sedentary time were observed between IG2 and IG1, in favor of IG2. In depth analyses indicated that MVPA increased at follow-up in participants who were male, aged <70 years, had a high level of education, and reached the WHO recommendations for MVPA at baseline (but the result was not significant).	The addition of an accelerometer to a web-based intervention for physical activity promotion among older adults may be an effective strategy to enhance physical activity in this population.
Paul, 2017 - Journal of Rehabilitation and Assistive Technologies Engineering	a. To map and describe the operationalisation of behaviour change techniques within the STARFISH app.<br>b. To explore user experience in terms of the acceptability and usability of the STARFISH app<br>c. To demonstrate the app’s potential to support older adults in increasing their daily step counts over a six	Convenience sampling from the University of the Third Age, Glasgow Seniors Forum, the University of Glasgow, and family/friends of the research team.	Participants were provided with an accelerometer-equipped smartphone (Samsung Galaxy SII) to record step counts. Participants could see in the smartphone home screen wallpaper (STARFISH app) the step count target and the four personal fish avatars of his/hers intervention group. The fins and tail of their fish avatar grow as the user approaches their target, which for the first week was set by adding 10% to the baseline. At the end of each week thereafter, if users had achieved their daily step count target on 5/7 days their target for the following week was increased by 10%, otherwise it remained unchanged. If all four members reach their weekly target the group is rewarded by the addition of a sea creature. The size of the fish fins and tail were reset each week, but any sea creature rewards the group	The mean daily step count increased by 14% (from 9,443 ± 3,952 steps before the intervention to 10,773 ± 2,659 steps after the intervention). The effect size was moderate (d=0.56), but not statistically significant (p=0.077). Generally, participants with lower baseline step counts showed larger increases than those with relatively higher baseline step counts.	The STARFISH app with group goals and rewards could be a feasible and effective tool in increasing physical activity levels of adults.

Publication	Study aim	Recruitment	Description	Results	Conclusion
	week period.		had received remained in the tank for the duration of the intervention. STARFISH also contains a personal planner that prompts users to make specific plans on how they are going to reach their step count target each day.		
Pischke, 2022 - JMIR mHealth and uHealth	To evaluate the feasibility and effectiveness of Web-based and print-based interventions for increasing moderate-to-vigorous physical activity (MVPA) and reducing sedentary behavior (SB) in older adults.	Active selection from 14 districts in Bremen, Germany, where the project team had already established previous liaisons (that were not included in the PROMOTE I study and/or in close proximity to the two study centers) and passive recruitment thanks to newspaper advertisements and talks of the research staff.	Depending on the group assignment (WEB intervention group, a WEB+ intervention group, and a PRINT control group), participants were provided with brochures outlining exercises for different difficulty levels and gender and a diary to track PA either in printed form or available on the website. The smartphone app additionally provided access to the exercises and PA diary for individuals in the WEB and WEB+. The WEB+ group also recieved a PA tracker (Fitbit Zip) synchronized with the website to objectively track daily step count. Weekly feedback regarding PA performed and goals achieved was provided either on the website, in the Android web app, or in the printed diary. In conjunction with the 10-week intervention, all 3 groups were offered weekly 90-minute face-to-face group sessions to practice and discuss in groups. Group meetings were continued monthly for another 6 months, after which participants chose whether to start using material from one of the other groups. The intervention content was based on self-regulation theory and various behavior change techniques facilitating regular self-monitoring of PA.	No significant differences in efficacy were observed across the 3 intervention groups. After 3 months, 80.6% (195/242) of the participants completed T1, and 66.1% (160/242) of participants completed T2. Of the participants who completed T2, attrition rates from baseline were 33.9%.	The three interventions (WEB, WEB+, and PRINT) were similar in feasibility and effectiveness in increasing MVPA and reducing SB levels among participants.
Roh, 2022 - PLOSONE	To assess the effectiveness of a motivational enhancement smartphone application in promoting brain	Participants were recruited from the outpatient clinic of Ajou University Hospital, Republic of Korea, with 49 participants randomized into either	Both groups received an educational session on brain health behaviors and a manual detailing physical, cognitive and dietary activities. Once a week, the smartphone app sent a push alert to intervention group participants to watch a video of the "family-coach" (family, friends, medical staff, case manager) offering support to reinforce motivation.	The intervention group showed a positive association with moderate metabolic equivalent activities and intrinsic motivation change. There was a significant difference between the intervention and control groups in average physical activity at week 8.	The motivational enhancement smartphone application was effective in inducing physical activity of moderate intensity through intrinsic

Publication	Study aim	Recruitment	Description	Results	Conclusion
	health behavior through lifestyle improvements, including physical activity, cognitive activity, and a healthy diet.	the experimental or control group.	Second, intervention group participants could rate their commitment to brain health behaviors and track weekly self-assessed outcomes through the app, comparing their achievments from one week to the next.		motivation enhancement.
Taraldsen, 2020 - Frontiers in Digital Health	a. To test the feasibility of aLiFE or eLiFE programme compared to general written physical activity recommendations only on young seniors aged 61–70 years.<br>b. To evaluate and further improve the intervention for a future phase III clinical trial, addressing participation and adherence, feasibility and usability, acceptability of eLiFE delivered using smartphone and smartwatch technology, estimates of change in function and physical activity, and feasibility of health economics	Via invitation letters sent to a random sample of individuals born between 01/01/1947 and 31/12/1956, drawn from the respective local population registries (Trondheim, Stuttgart, Amsterdam).	The two intervention groups (eLife and aLife) received a guided activity plan. The eLiFE participants were provided with both an android phone and a smartwatch they could use to access the PreventIT application, which included video clips, pictures, and text/verbal instructions for each activity. The aLiFE participants received a paper-based manual with descriptions and instructions for the same activities. Participants in aLiFE and eLiFE received six and four home visits from trainers, respectively, plus three phone-calls. After the 6 month active intervention period, participants were encouraged to continue with their personalized activity programme for the next 6 months, without the assistance from trainers (unsupervised follow-up). The control group participants only received one home visit entailing a two-page written summary of the WHO recommendations for physical activity. The intervention programme consisted of strategies to (a) improve balance, (b) increase muscle strength, and (c) reduce sedentariness and increase physical activity. The aLiFE programme was adapted to young seniors to be more challenging than the LiFE programme.	No significant differences between the three groups have been found for the primary outcome measures, LLFDI, physical behavior complexity metric ($p > 0.01$), or secondary outcomes ($p > 0.01$). For several of the outcomes, all groups showed improvements over time. During the intervention period, 47 participants reported full adherence (16 eLiFE, 11 aLiFE, 20 controls), 88 reported partial adherence (28 eLiFE, 32 aLiFE, 28 controls), and 29 were non-adherent to their intervention (10 eLiFE, 12 aLiFE, 7 controls). During the unsupervised follow-up period, 34 participants reported full adherence (10 eLiFE, 7 aLiFE, 17 controls), 73 reported partial adherences (26 eLiFE, 25 aLiFE, 22 controls), and 40 were non-adherent to their intervention (10 eLiFE, 20 aLiFE, 10 controls). eLiFE participants tended to select fewer activities (9.1 reported activities, SD 5.1) but at more challenging levels, while aLiFE participants trained at all levels of difficulty (10.0 activities, SD 5.8) activities. In both intervention groups, the “one-leg stand” was the most reported activity (n = 67) followed by “stair climbing” and “tandem walk” (both n = 58), and the least reported activity was square jumping on one leg (n = 4).	All three groups improved in some of the clinical outcome measures over time, suggesting that improved function and behavioral change can be achieved in different ways. Combining technology with behavior change techniques, was as successful as delivering the intervention using traditional paper-based manuals. Participants were satisfied with both the technology-based version and the traditional delivered paper-based version, but the “human element” of involving an instructor was valued as highly motivating.

Publication	Study aim	Recruitment	Description	Results	Conclusion
	evaluation.				
Wijsman, 2013 - Journal of Medical Internet Research	a. To test the effectivenes in 60-70 year old inactive individuals of a 3-months Internet program which focused on effective components of health behavior change including self-monitoring by accelerometer and goal setting with the help of digital coaching .<br>b. To test the effect of this intervention on metabolic health including anthropometric measures and markers of glucose and lipid metabolism.	Through advertisement in local newspapers and press notification, directing participants motivated to increase physical activity to the study website, where they could complete an online questionnaire.	An internet-based interactive coaching program personalized the targets for daily activity for each participant as the absolute increase in physical activity compared to the individual's baseline assessment data (approximately 10% in daily physical activity at week 12, at a linear rate per week). All participants were given the option to decrease or increase the personalized goal, within limits. No specific instructions regarding daily physical activity were given to the delayed control group.<br>Three elements were provided to participants in the intervention group: (1) an accelerometer-based activity monitor, (2) a personal website, and (3) a personal e-coach, who provides regular updates of the individual's physical activity status by email and gives advice on how to increase physical activity. DirectLife intervention is based on the stages of change and the I-Change model, with the aim to increase awareness, give feedback, and provide support to make sustainable changes in physical activity behavior.	After 13 weeks, intervention group participants increased their daily physical activity measured at the ankle by 46% and measured by the wrist accelerometer by 11%, compared to 12% (Pdifference<.001) and 5% (Pdifference=.11), respectively, in the control group. In the intervention group, there was a mean increase of 11.1 minutes per day spent in MVPA, compared to a mean decrease of 0.1 minutes in the control group (Pdifference=.001). Weight, waist circumference, fat percentage, HbA1c, glucose metabolism, fasting insulin levels improved significantly more in the intervention vs control group, as did the HOMA-index. Total cholesterol, LDL, and triglycerides levels improved in the intervention group, but these differences were not significant between groups. Systolic blood pressure and resting heart rate improved in both groups. No significant change in grip strength was seen in either of the groups.	The web-based intervention was effective in increasing daily physical activity and improving metabolic health in inactive elderly after 3 months. The intervention was feasible for use in an older population, with high retention rates.
