# Supplementary material for: Digital Health Interventions to Promote Physical Activity in Community-Dwelling Older Adults: A Systematic Review and Semiquantitative Analysis
Source: Int J Public Health. 2025 Jan 3;69:1607720. doi: 10.3389/ijph.2024.1607720 (PMC11738617; doi:10.3389/ijph.2024.1607720)
Supplement: Supplementary file 3 [file DataSheet6.pdf]

Supplementary file 6. Abbreviations

Acronyms

AI	Artificial Intelligence
App	Application
ChatGPT-4	Chat Generative Pre-Trained Transformer-4
DHI	Digital Health Intervention
Ehealth	Electronic Health
FDA	Food And Drug Administration
HbA1c	Glycated Hemoglobin
IT	Information Technology
mHealth	Mobile Health
MVPA	Moderate-to-Vigorous Physical Activity
OA	Older Adults
PA	Physical Activity
PRISMA	Preferred Reporting Items For Systematic Review And Meta-Analyses
QoL	Quality Of Life
RCT	Randomized Controlled Trial
WHO	World Health Organization

Study identification

Alley, 2022	Alley SJ, Uffelen J van, Schoeppe S, Parkinson L, Hunt S, Power D, et al. The Effectiveness of a Computer-Tailored Web-Based Physical Activity Intervention Using Fitbit Activity Trackers in Older Adults (Active for Life): Randomized Controlled Trial. Journal of Medical Internet Research. 2022 May 12;24(5):e31352
Cai, 2022	Cai X, Qiu S, Luo D, Li R, Liu C, Lu Y, et al. Effects of peer support and mobile application-based walking programme on physical activity and physical function in rural older adults: a cluster randomized controlled trial. Eur Geriatr Med. 2022 Oct 1;13(5):1187–95

Compernolle, 2020	Compernolle S, Cardon G, Ploeg HP van der, Nassau FV, Bourdeaudhuij ID, Jelsma JJ, et al. Engagement, Acceptability, Usability, and Preliminary Efficacy of a Self-Monitoring Mobile Health Intervention to Reduce Sedentary Behavior in Belgian Older Adults: Mixed Methods Study. JMIR mHealth and uHealth. 2020 Oct 29;8(10):e18653
Granet, 2023	Granet J, Peyrusqué E, Ruiz F, Buckinx F, Abdelkader LB, Dang-Vu TT, et al. Web-Based Physical Activity Interventions Are Feasible and Beneficial Solutions to Prevent Physical and Mental Health Declines in Community-Dwelling Older Adults During Isolation Periods. The Journals of Gerontology: Series A. 2023 Mar 1;78(3):535–44
Kim, 2013	Kim BH, Glanz K. Text Messaging to Motivate Walking in Older African Americans: A Randomized Controlled Trial. American Journal of Preventive Medicine. 2013 Jan 1;44(1):71–5
Mendoza-Vasconez, 2024.	Mendoza-Vasconez AS, King AC, Chandler G, Mackey S, Follis S, Stefanick ML. Engagement With Remote Delivery Channels in a Physical Activity Intervention for Senior Women in the US. Am J Health Promot. 2024 Jun 1;38(5):692–703
Muellmann, 2019	Muellmann S, Buck C, Voelcker-Rehage C, Bragina I, Lippke S, Meyer J, et al. Effects of two web-based interventions promoting physical activity among older adults compared to a delayed intervention control group in Northwestern Germany: Results of the PROMOTE community-based intervention trial. Preventive Medicine Reports. 2019 Sep 1;15:100958
Paul, 2017	Paul L, Brewster S, Wyke S, McFadyen AK, Sattar N, Gill JM, et al. Increasing physical activity in older adults using STARFISH, an interactive smartphone application (app); a pilot study. Journal of Rehabilitation and Assistive Technologies Engineering. 2017 Jan 1;4
Pischke, 2022	Pischke CR, Voelcker-Rehage C, Ratz T, Peters M, Buck C, Meyer J, et al. Web-Based Versus Print-Based Physical Activity Intervention for Community-Dwelling Older Adults: Crossover Randomized Trial. JMIR mHealth and uHealth. 2022 Mar 23;10(3):e32212

Roh, 2022	Roh HW, Ryu H, Jeong S, Han J, Park B, Moon SY, et al. The effectiveness of a motivational enhancement smartphone application promoting lifestyle improvement for brain health: A randomized controlled trial. Farrukh MJ, editor. PLoS ONE. 2022 Jun 30;17(6):e0267806
Taraldsen, 2020	Taraldsen K, Mikolaizak AS, Maier AB, Mellone S, Boulton E, Aminian K, et al. Digital Technology to Deliver a Lifestyle-Integrated Exercise Intervention in Young Seniors—The PreventIT Feasibility Randomized Controlled Trial. Front Digit Health. 2020 Jul 31;2:10
Wijsman, 2013	Wijsman CA, Westendorp RG, Verhagen EA, Catt M, Slagboom PE, Craen AJ de, et al. Effects of a Web-Based Intervention on Physical Activity and Metabolism in Older Adults: Randomized Controlled Trial. Journal of Medical Internet Research. 2013 Nov 6;15(11):e2843
