# Supplementary material for: Digital Health Interventions to Promote Physical Activity in Community-Dwelling Older Adults: A Systematic Review and Semiquantitative Analysis
Source: Int J Public Health. 2025 Jan 3;69:1607720. doi: 10.3389/ijph.2024.1607720 (PMC11738617; doi:10.3389/ijph.2024.1607720)
Supplement: Supplementary file 4 [file DataSheet3.pdf]

Supplementary file 3. Detail of intervention tools

Overview of the main intervention tools adopted in each study

Tools and features		*Alley et al.*	*Cai et al.*	*Compernolle et al.*	*Granet et al.*	*Kim et al.*	*Mendoza - vasconez et al.*	*Muellmann et al.*	*Paul et al.*	*Pischke et al.*	*Roh et al.*	*Taraldsen et al.*	*Wijsman et al.*
**Wearable tracker**		Yes	Yes	Yes	No	Yes	Yes	Yes	Yes	Yes	No	No	Yes
**Personal physical activity diary**	*Synchronized with wearable*	Yes	No	Yes	No	No	No	Yes	Yes	Yes	No	No	Yes
	Manual filling in	No	Yes	No	No	Yes	Yes	No	No	No	Yes	No	No
**Goal tracking interface**	*With goals set from the start*	Yes	Yes	Yes	Yes	No	No	Yes	Yes	Yes	No	Yes	Yes
	With adaptive goals	Yes	No	No	No	No	No	No	Yes	No	No	No	Yes
	With digital rewards	No	No	No	No	No	No	Yes	Yes	No	No	No	No
**Instructions for exercises**	*Pre-recorded videos*	No	No	No	Yes	No	Yes*	No	No	No	No	Yes	No
	Written brochures	No	Yes	No	No	No	Yes	Yes	No	Yes	Yes	Yes	No
**Communication platform**	*Motivational messages*	No	No	No	No	Yes	Yes	Yes	No	No	Yes	No	No
	Support from peers	No	Yes	No	No	No	No	Yes*	Yes	No	Yes	No	No
**Human technical assistance**	*At the start of the intervention*	No	Yes	Yes	No	No	No	No	No	No	Yes	No	No
	All along	No	Yes	No	No	Yes*	Yes*	Yes*	No	Yes	No	Yes	No

** the use of these tools was elective in the studies, the use of other tools was instead mandatory in the other studies*
