# Supplementary material for: Digital Health Interventions to Promote Physical Activity in Community-Dwelling Older Adults: A Systematic Review and Semiquantitative Analysis
Source: Int J Public Health. 2025 Jan 3;69:1607720. doi: 10.3389/ijph.2024.1607720 (PMC11738617; doi:10.3389/ijph.2024.1607720)
Supplement: Supplementary file 5 [file DataSheet1.PDF]

Supplementary file 1. Search strings

Pubmed

#1 Search: (("old people"[Title/Abstract]) OR ("older people"[Title/Abstract]) OR ("old adult"[Title/Abstract]) OR ("older adult"[Title/Abstract]) OR ("old adults"[Title/Abstract]) OR ("older adults"[Title/Abstract]) OR ("old population"[Title/Abstract]) OR ("older population"[Title/Abstract]) OR ("old age"[Title/Abstract]) OR ("older age"[Title/Abstract]) OR ("elder"[Title/Abstract]) OR ("elderly"[Title/Abstract]) OR ("senior"[Title/Abstract]) OR ("geriatric"[Title/Abstract]) OR ("geriatrics"[Title/Abstract]) OR ("aging adult"[Title/Abstract]) OR ("aging adults"[Title/Abstract]) OR ("aging people"[Title/Abstract]) OR ("aging population"[Title/Abstract]) OR ("ageing adult"[Title/Abstract]) OR ("ageing adults"[Title/Abstract]) OR ("ageing people"[Title/Abstract]) OR ("ageing population"[Title/Abstract]) OR ("60 years"[Title/Abstract]) OR ("aged 60"[Title/Abstract]))

#2 Search: (("Mobile Health"[Title/Abstract]) OR ("Telehealth"[Title/Abstract]) OR ("mHealth"[Title/Abstract]) OR ("eHealth"[Title/Abstract]) OR ("m-health"[Title/Abstract]) OR ("e-health"[Title/Abstract]) OR ("SMS"[Title/Abstract]) OR ("MMS"[Title/Abstract]) OR ("Messaging"[Title/Abstract]) OR ("Texting"[Title/Abstract]) OR ("Text Message"[Title/Abstract]) OR ("Text Messages"[Title/Abstract]) OR ("Electronic Mail"[Title/Abstract]) OR ("Email"[Title/Abstract]) OR ("E-Mail"[Title/Abstract]) OR ("Electronic Mails"[Title/Abstract]) OR ("Emails"[Title/Abstract]) OR ("E-Mails"[Title/Abstract]) OR ("Mobile Application"[Title/Abstract]) OR ("Mobile Applications"[Title/Abstract]) OR ("Mobile Apps"[Title/Abstract]) OR ("Mobile App"[Title/Abstract]) OR ("Smartphone Apps"[Title/Abstract]) OR ("Smartphone App"[Title/Abstract]) OR ("iOS Apps"[Title/Abstract]) OR ("iOS App"[Title/Abstract]) OR ("Android Apps"[Title/Abstract]) OR ("Android App"[Title/Abstract]) OR ("Internet-Based"[Title/Abstract]) OR ("Web-Based"[Title/Abstract]) OR ("Phone Call"[Title/Abstract]) OR ("Telephone Call"[Title/Abstract]) OR ("Automated Call"[Title/Abstract]) OR ("Automated notification"[Title/Abstract]) OR ("Phone Calls"[Title/Abstract]) OR ("Telephone Calls"[Title/Abstract]) OR ("Automated Calls"[Title/Abstract]) OR ("Automated notifications"[Title/Abstract]))

#3 Search: (("Sedentary"[Title/Abstract]) OR ("Inactive"[Title/Abstract]) OR ("Inactivity"[Title/Abstract]) OR ("Active lifestyle"[Title/Abstract]) OR ("Active life"[Title/Abstract]) OR ("Active ageing"[Title/Abstract]) OR ("Active aging"[Title/Abstract]) OR ("Physical"[Title/Abstract]) OR ("Sport"[Title/Abstract]) OR ("Sports"[Title/Abstract]) OR ("Aerobic"[Title/Abstract]) OR ("Aerobics"[Title/Abstract]) OR ("Gym"[Title/Abstract]) OR ("Fitness"[Title/Abstract]) OR ("Walk"[Title/Abstract]) OR ("Walking"[Title/Abstract]) OR ("Exercise"[Title/Abstract]) OR ("Exercises"[Title/Abstract]) OR ("Stretching"[Title/Abstract]) OR ("Workout"[Title/Abstract]) OR ("Motor Activity"[Title/Abstract]) OR ("Motor Activities"[Title/Abstract]))

#4 Search: (review[Publication Type] OR systematic review[Publication Type] OR meta-analysis[Publication Type])

#5 Search: #1 AND #2 AND #3 NOT #4 Filters: English

Web of Science

("old people" OR "older people" OR "old adult" OR "older adult" OR "old adults" OR "older adults" OR "old population" OR "older population" OR "old age" OR "older age" OR "elder" OR "elderly" OR "senior" OR "geriatric" OR "geriatrics" OR "aging adult" OR "aging adults" OR "aging people" OR "aging population" OR "ageing adult" OR "ageing adults" OR "ageing people" OR "ageing population" OR "60 years" OR "aged 60") (Topic)

AND ("Mobile Health" OR "Telehealth" OR "mHealth" OR "eHealth" OR "m-health" OR "e-health" OR "SMS" OR "MMS" OR "Messaging" OR "Texting" OR "Text Message" OR "Text Messages" OR "Electronic Mail" OR "Email" OR "E-Mail" OR "Electronic Mails" OR "Emails" OR "E-Mails" OR "Mobile Application" OR "Mobile Applications" OR "Mobile Apps" OR "Mobile App" OR "Smartphone Apps" OR "Smartphone App" OR "iOS Apps" OR "iOS App" OR "Android Apps" OR "Android App" OR "Internet-Based" OR "Web-Based" OR "Phone Call" OR "Telephone Call" OR "Automated Call" OR "Automated notification" OR "Phone Calls" OR "Telephone Calls" OR "Automated Calls" OR "Automated notifications") (Topic)

AND ("Sedentary" OR "Inactive" OR "Inactivity" OR "Active lifestyle" OR "Active life" OR "Active ageing" OR "Active aging" OR "Physical" OR "Sport" OR "Sports" OR "Aerobic" OR "Aerobics" OR "Gym" OR "Fitness" OR "Walk" OR "Walking" OR "Exercise" OR "Exercises" OR "Stretching" OR "Workout" OR "Motor Activity" OR "Motor Activities") (Topic)

AND English (Language)

NOT Review (Document Type)

Scopus

TITLE-ABS-KEY ("old people" OR "older people" OR "old adult" OR "older adult" OR "old adults" OR "older adults" OR "old population" OR "older population" OR "old age" OR "older age" OR "elder" OR "elderly" OR "senior" OR "geriatric" OR "geriatrics" OR "aging adult" OR "aging adults" OR "aging people" OR "aging population" OR "ageing adult" OR "ageing adults" OR "ageing people" OR "ageing population" OR "60 years" OR "aged 60")

AND TITLE-ABS-KEY ("Mobile Health" OR "Telehealth" OR "mHealth" OR "eHealth" OR "m-health" OR "e-health" OR "SMS" OR "MMS" OR "Messaging" OR "Texting" OR "Text Message" OR "Text Messages" OR "Electronic Mail" OR "Email" OR "E-Mail" OR "Electronic Mails" OR "Emails" OR "E-Mails" OR "Mobile Application" OR "Mobile Applications" OR "Mobile Apps" OR "Mobile App" OR "Smartphone Apps" OR "Smartphone App" OR "iOS Apps" OR "iOS App" OR "Android Apps" OR "Android App" OR "Internet-Based" OR "Web-Based" OR "Phone Call" OR "Telephone Call" OR "Automated Call" OR "Automated notification" OR "Phone Calls" OR "Telephone Calls" OR "Automated Calls" OR "Automated notifications")

AND TITLE-ABS-KEY ("Sedentary" OR "Inactive" OR "Inactivity" OR "Active lifestyle" OR "Active life" OR "Active ageing" OR "Active aging" OR "Physical" OR "Sport" OR "Sports" OR "Aerobic" OR "Aerobics" OR "Gym" OR "Fitness" OR "Walk" OR "Walking" OR "Exercise" OR "Exercises" OR "Stretching" OR "Workout" OR "Motor Activity" OR "Motor Activities")

AND (EXCLUDE (DOCTYPE , "re"))

AND (LIMIT-TO (LANGUAGE , "English"))
