# Supplementary material for: Digital Health Interventions to Promote Physical Activity in Community-Dwelling Older Adults: A Systematic Review and Semiquantitative Analysis
Source: Int J Public Health. 2025 Jan 3;69:1607720. doi: 10.3389/ijph.2024.1607720 (PMC11738617; doi:10.3389/ijph.2024.1607720)
Supplement: Supplementary file 6 [file DataSheet5.PDF]

Supplementary file 5. Secondary outcomes results

For Roh et al. (2022) no statistically significant difference on cognitive activity or healthy diet activity. For Mendoza-Vasconez et al. (2024), the highest engagement was reported with targeted print inserts (29%), while lower engagement was seen with IVR (interactive voice response) and website (around 1% for each). Conditional Inference Tree Analysis highlighted that the participants with higher initial PA, younger age, better physical functioning, and no need for mobility aids were more likely to engage. For Kim et al. (2022), participants maintain perceived physical activity levels after the lockdown, despite significant increases in sitting time among young and older adults. Decreases in moderate physical activity frequency were associated with a higher level of depressive symptoms (R^2 = 17.1%). For Taraldsen et al. (2020) the intervention group showed a positive association with moderate MET activities after adjusting for age, sex, education years, K-MMSE score, SGDS-K score, and BFI-K-10 score (p = 0.01) and with intrinsic motivation change after adjusting for age, sex, education years, K-MMSE score, SGDS-K score, and BFI-K-10 score (p = 0.01). Also adherence was higher in the intervention group. Granet et al. 2023, reports similar adherence rates (live group LG: 89%; recorded group RG: 81%), level of satisfaction (LG: 77% vs RG: 64%), and enjoyment (LG: 68% vs RG: 62%) between groups, however different the perceived difficulty (LG: 58% vs RG: 63%). Both groups significantly improved on functional capacities, physical performance, and quality of life. Only the LG showed significant improvements in perceived health and PA level. The LG showed greater improvements in physical performance and quality of life than the RG. For Alley et al. (2022), participants in the "tailoring+Fitbit" group reported significant reductions in daily sitting time at 12 weeks relative to the control group, however discordant with objectively registered data. Neither BMI or other parameters reported significant differences. For Wijsman et al. (2013), weight decreased significantly more in the intervention group compared to controls (−1.5 kg vs −0.8 kg respectively, p=0.046), as did waist circumference (−2.3 cm vs −1.3 cm

respectively, $p=0.036$) and fat mass (−0.6% vs 0.07% respectively, $p=0.025$). Insulin and HbA1c levels were significantly more reduced in the intervention group ($p<0.05$). Muellman et al. (2019) reported a 71% attendance to weekly meetings, group exercises were rated highly useful (56% found balance training helpful) while health education components were poorly rated (only 19% found social support information helpful). For Compernolle et al. (2020), participants mainly reported positive feelings, with a thinking change rather than actual behavior change. There were mixed opinions on the kind of feedback (ie, tactile vs visual) that they preferred. The intervention was considered easy to use with a clear design. Some problems were noticed regarding attaching and wearing the self-monitoring device. The median frequency of consulting the app widely differed among participants (0 to 20 times a day). For Cai et al. (2022), no significant outcomes were observed on gait speed, chair-rising time, or body composition. For Pischke et al. (2022) the intervention acceptance was generally high. The use of intervention material was high to moderate at first and decreased during the follow-up. Paul et al. (2017) report good experiences from the app users.
